# A Step-By-Step Guide for Robotic Blumgart Pancreaticojejunostomy

**DOI:** 10.3390/jcm14134471

**Published:** 2025-06-24

**Authors:** Siyuan Qian, Jeison Carrillo-Peña, Víctor Domínguez-Prieto, Pedro Villarejo-Campos, Montiel Jiménez-Fuertes, Pablo Pastor-Riquelme, Santos Jiménez-Galanes

**Affiliations:** 1 Surgery Department, Fundación Jiménez Díaz University Hospital, Avda. Reyes Católicos, 2, 28040 Madrid, Spain; 2 Surgery Department, Faculty of Medicine, Universidad Autónoma de Madrid, 28029 Madrid, Spain

**Keywords:** Blumgart anastomosis, robotic pancreaticoduodenectomy, robotic pancreaticojejunostomy

## Abstract

**Background:** In recent years, the use of minimally invasive approaches in pancreatic surgery has progressively increased. One of the key components of pancreaticoduodenectomy is the creation of a pancreato-enteric anastomosis, due to the high risk of postoperative complications, particularly the development of postoperative pancreatic fistula. Among the types of anastomoses, the Blumgart technique has gained popularity due to its ease of reproducibility. **Methods:** In this guide, we summarize and systematize step by step how to perform a feasible, reproductible and safe robotic Blumgart pancreaticojejunostomy, providing some instructions for its successful completion. **Results:** Despite the heterogeneity of the published data, duct-to-mucosa Blumgart anastomosis seems to be superior in terms of clinically relevant postoperative pancreatic fistula rates compared with other types of pancreato-enteric anastomosis. The advantages of robotic surgery, such as improved precision, greater control, and enhanced visualization, make robotic Blumgart anastomosis a safe, practical, and reproducible technique in the context of robotic pancreaticoduodenectomy. **Conclusions:** Robotic Blumgart pancreaticojejunostomy is a safe and feasible technique for pancreato-enteric anastomosis following pancreaticoduodenectomy when surgical technique is systematized step by step.

## 1. Introduction

It is well known that periampullary lesions and pancreatic head neoplasms are often indicated for pancreaticoduodenectomy (PD). In recent years, the use of minimally invasive approaches in pancreatic surgery has progressively increased [1]. The 2023 International Consensus Guidelines on Robotic Pancreatic Surgery establish robotic pancreaticoduodenectomy (RPD) as an indication for both benign and malignant tumors of the pancreatic head and periampullary region, as well as for borderline resectable tumors of the pancreatic head requiring PD [2]. Open pancreaticoduodenectomy (OPD), laparoscopic PD, and RPD appear to have similar safety profiles. However, when comparing these three techniques, the use of laparoscopic and robotic surgery is associated with a lower risk of infectious and pulmonary complications, fewer overall complications, reduced blood loss, less postoperative bleeding, shorter hospital stays, and a decreased rate of hospital readmissions [3,4].

One of the key components of PD is the creation of a pancreato-enteric anastomosis, due to the high risk of postoperative complications, particularly the development of a postoperative pancreatic fistula (POPF). Numerous studies have explored different surgical techniques aimed at reducing the incidence of clinically relevant postoperative pancreatic fistula (CR-POPF) [5]. Pancreaticojejunostomy (PJ) remains the preferred method globally, with a reported preference rate of 88.7% of cases [6]. Alternatively, some groups prefer to perform a pancreaticogastrostomy (PG), though this is less common due to its higher risk of bleeding [7]. Due to the lack of evidence supporting the superiority of one technique over others, there is currently no international consensus for reconstructing the pancreatic stump after PD, so pancreatic anastomosis remains a topic of active debate and the technique to be performed is usually a personal decision of each surgeon [8]. Concerning PJ, numerous techniques have been proposed as a way to minimize the risk of POPF [9]. Recently, a technique approach to duct-to-mucosa anastomosis, known as Blumgart anastomosis (BA), has gained increasing popularity [10]. Some studies suggest that BA is not inferior to conventional anastomosis and may even be superior in reducing the number of complications [11,12].

## 2. Materials and Methods

In this guide and Supplementary Video S1, we summarize and systematize step by step how to perform a feasible, reproductible and safe robotic Blumgart pancreaticojejunostomy, providing some tips for its successful completion.

## 3. Results

**Figure 1:**
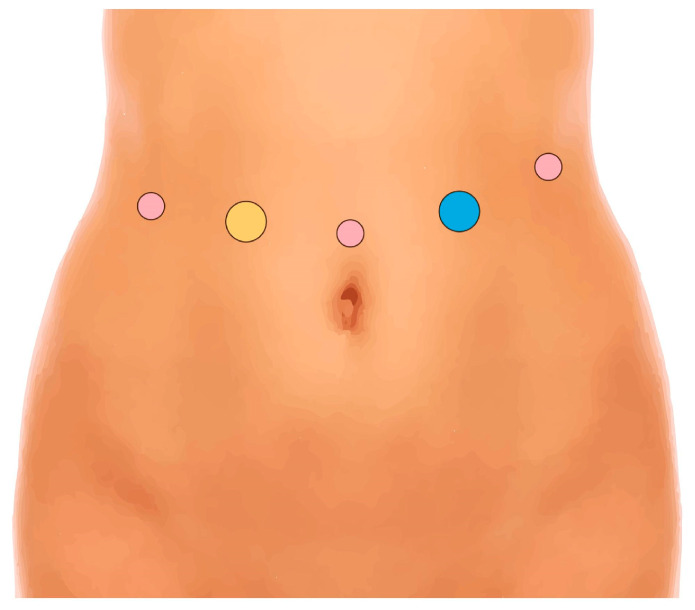
Position of robotic trocars. Yellow: assistant 12 mm trocar; pink: robotic 8 mm trocars; blue: robotic 12 mm trocar.

The patient, under general anesthesia, is positioned supine. Pneumoperitoneum is established using a Veress needle on the left hypochondrium (Palmer’s point). Robotic trocars are placed as shown (Figure 1).

**Figure 2:**
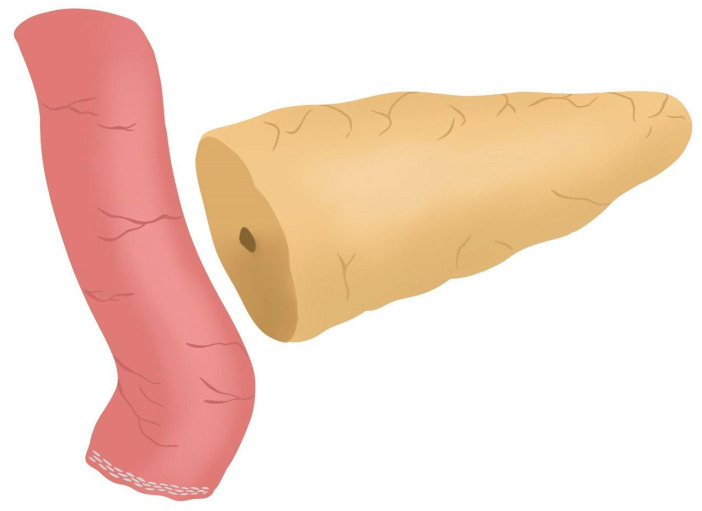
The biliopancreatic limb is placed facing the pancreatic stump.

**Figure 3:**
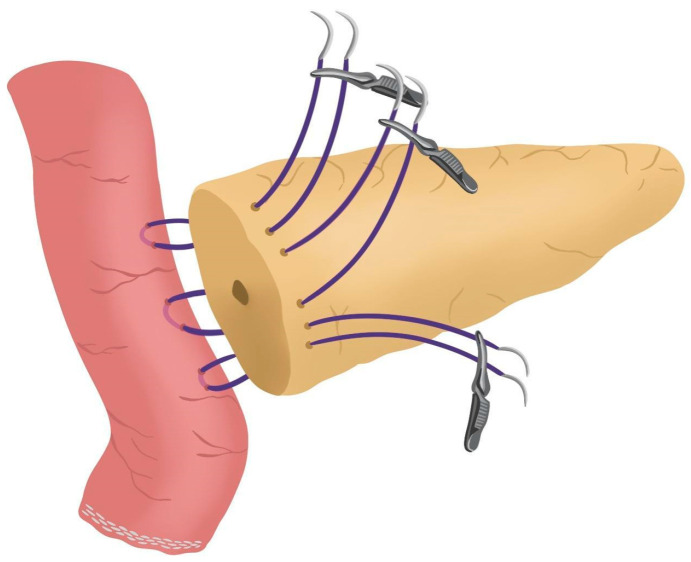
Posterior Blumgart stitches.

**Figure 4:**
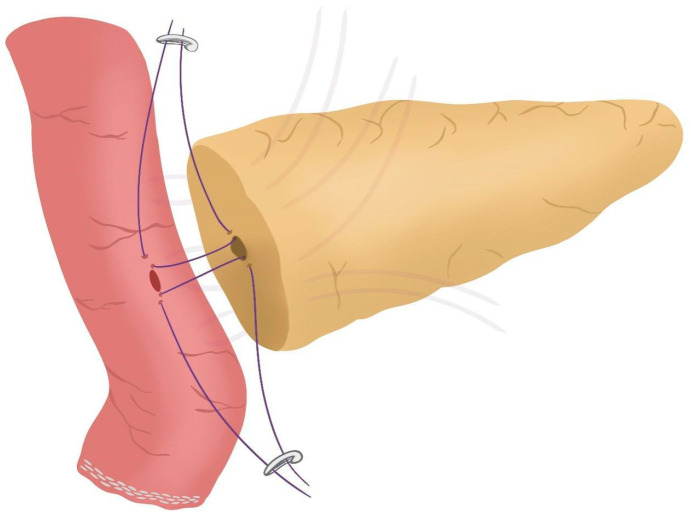
Corner stitches of the duct-to-jejunal anastomosis.

**Figure 5:**
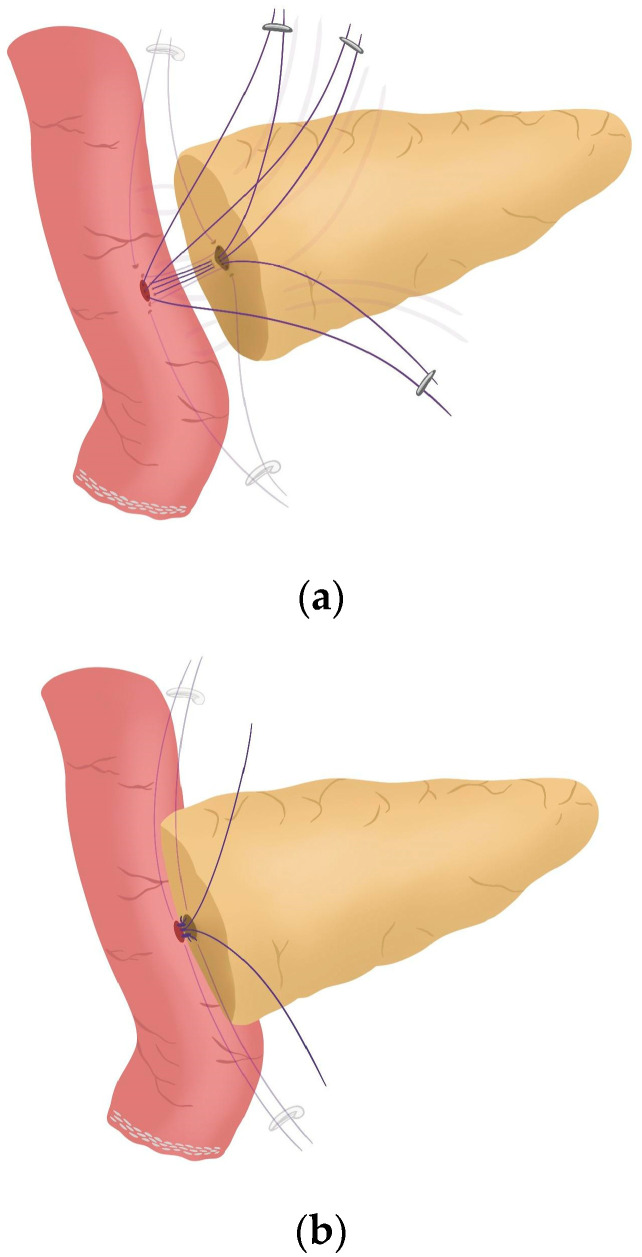
(**a**) Posterior wall stitches of the duct-to-mucosa anastomosis; (**b**) Tied posterior wall stitches of the duct-to-mucosa anastomosis.

**Figure 6:**
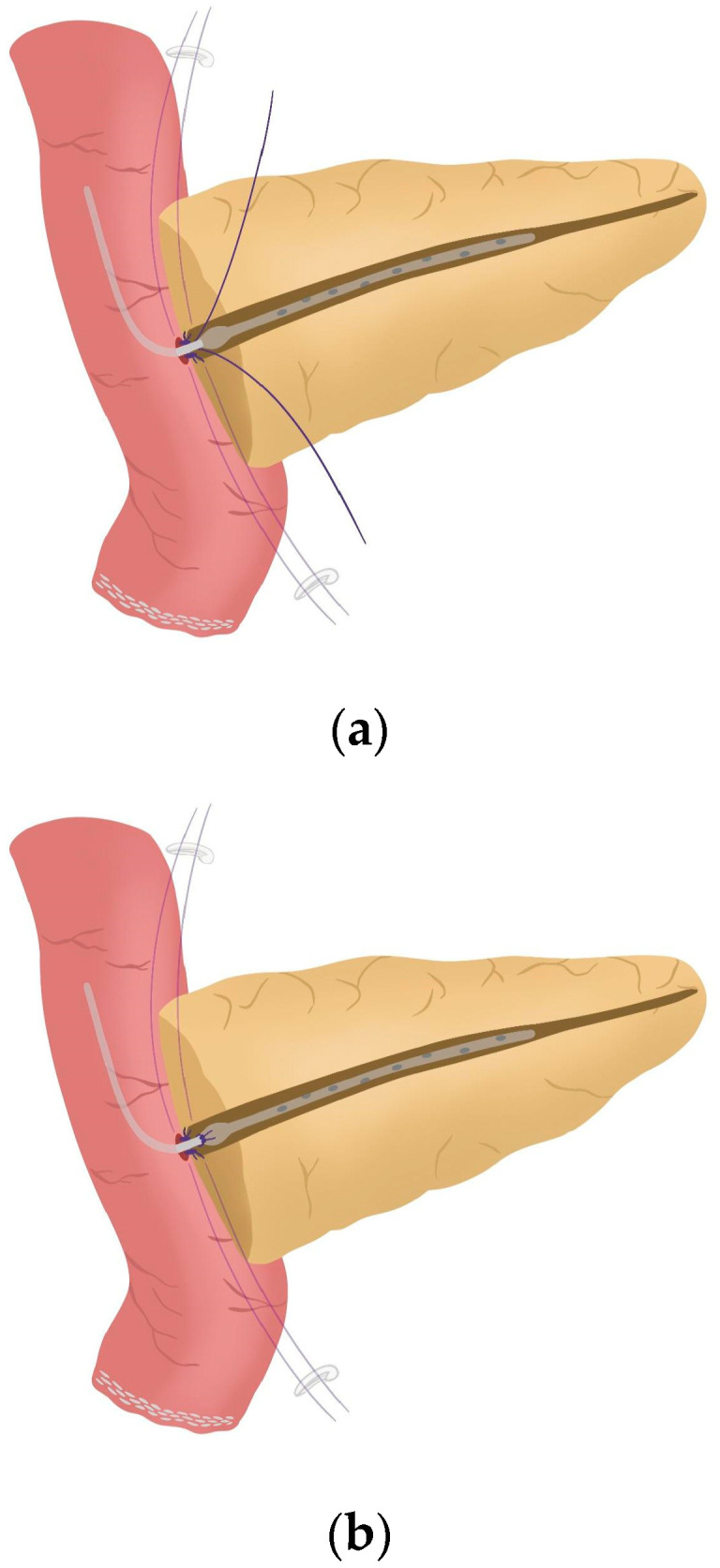
(**a**) Pancreatic duct drainage placed within the pancreatic duct and the biliopancreatic limb; (**b**) Pancreatic duct drainage is fixed by tying the central stitch of the posterior wall of the duct-to-mucosa anastomosis.

**Figure 7:**
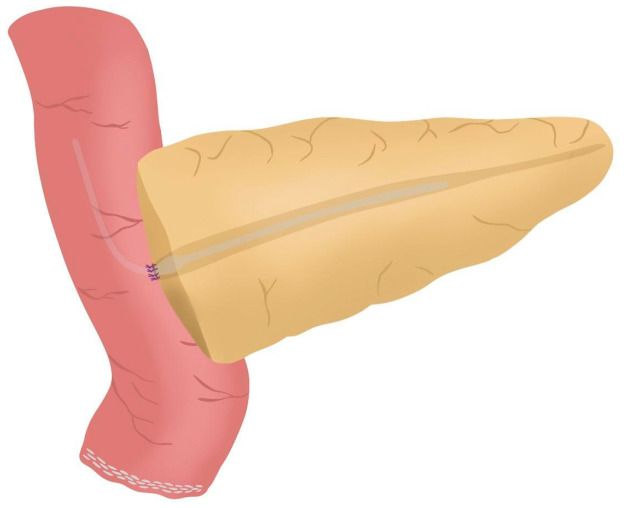
Anterior wall stitches are performed, completing the duct-to-mucosa anastomosis.

**Figure 8:**
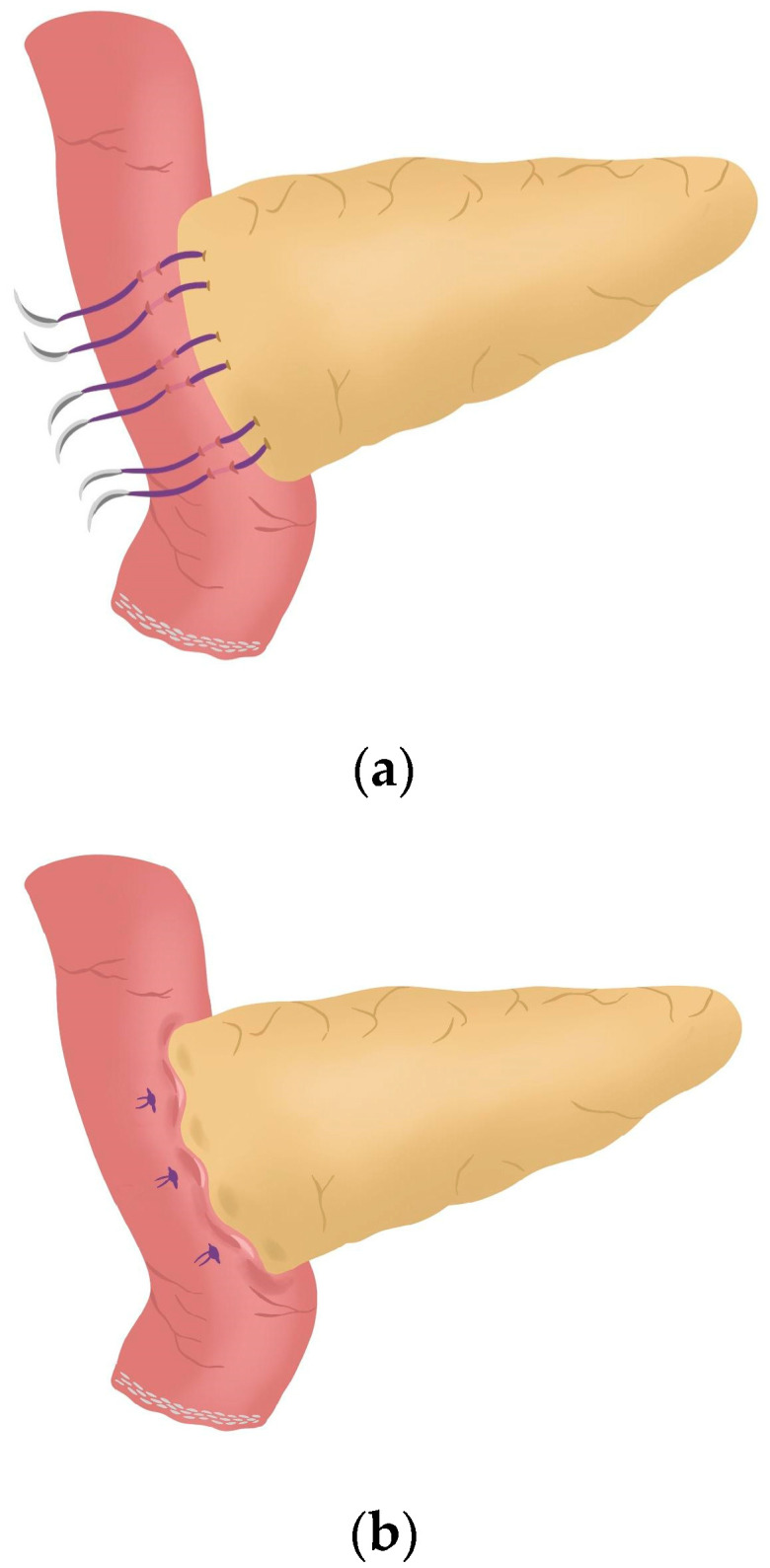
(**a**) Completion of Blumgart anastomosis; (**b**) The pancreatic stump remains invaginated against the jejunal serosa.

After completing the resection phase of the RPD, a PJ following the robot-assisted end-to-side Blumgart PJ is systematically performed:

1. The jejunal stump is marked with a simple stitch of 3/0 polydioxanone monofilament (see 00:20 in Supplementary Video S1).
2. The marked biliopancreatic limb is uncrossed through the duodenal tunnel and placed facing the pancreatic stump (Figure 2) (see 00:33 in Supplementary Video S1).
3. The length of the pancreatic duct drainage is tested. We use a multi-perforated 1–2 mm polyvinyl chloride pancreatic drainage, depending on the diameter of the pancreatic duct. It must fill the whole length of the remaining pancreatic duct (see 00:43 in Supplementary Video S1).
4. Posterior Blumgart stitches with a double-needle 3/0 polydioxanone monofilament are performed. We usually perform 3 Blumgart stitches, but it can vary depending on the size of the pancreas. The first of them (the lower one) is performed below the pancreatic duct; the second one (the central one) is usually performed surrounding the pancreatic duct (one stitch below and the other over the pancreatic duct, without damaging it); and the third one (the upper one) over the pancreatic duct. The needle is inserted through the full thickness of the pancreatic stump with each stitch, starting from the anterior face and exiting through the posterior face. After that, the same needle is stitched on the posterior wall of the jejunal serosa in a longitudinal direction. Finally, the entire thickness of the pancreatic stump is pierced again from its posterior to its anterior face, in an upper position. Each Blumgart stitch is marked with a bulldog clamp and the needles are stuck in an orderly manner in a wrapped gauze (Figure 3) (see 00:52 in Supplementary Video S1).
5. Prior to enterotomy, both side corner stitches of the duct-to-jejunal anastomosis are performed with a 5/0 polydioxanone monofilament and marked with Hem-o-locks^®^ (Figure 4) (see 01:36 in Supplementary Video S1).
6. Enterotomy in the antimesenteric side of the biliopancreatic limb is performed (see 02:10 in Supplementary Video S1).
7. Posterior wall stitches of the duct-to-mucosa anastomosis PJ are performed with 5/0 polydioxanone monofilament. They are marked with metallic clips to distinguish them from the corner stitches (marked with Hem-o-locks®) and subsequently tied when all the stitches have been performed (Figure 5) (see 02:16 in Supplementary Video S1).
8. The pancreatic duct drainage is definitively placed into the pancreatic duct and biliopancreatic limb. The central stitch of the posterior wall of the duct-to-mucosa anastomosis is tied fixing the pancreatic duct drainage (Figure 6). This pancreatic duct drainage stent has an olive that acts as a stopper, preventing migration and ensuring it remains in the desired position for at least one month (see 03:24 in Supplementary Video S1). In patients with soft pancreatic texture and/or a small pancreatic duct (≤3 mm), we employ an externalized pancreatic duct drainage by Witzel technique to minimize the risk of POPF.
9. Afterwards, the anterior wall stitches are performed with 5/0 polydioxanone monofilament, marked with metallic clips and subsequently tied, completing the duct-to-mucosa anastomosis (Figure 7) (see 04:02 in Supplementary Video S1).
10. Completion of Blumgart anastomosis: Finally, bulldog clamps are removed, and each needle of each double-needle 3/0 polydioxanone monofilament is stitched on the anterior wall of the jejunal serosa in a transverse direction and an orderly way (Figure 8a). Each of the three stitches are tied so that the pancreatic stump keeps invaginated against the jejunal serosa (Figure 8b) (see 04:39 in Supplementary Video S1).

## 4. Discussion

Since POPF remains one of the primary causes of morbidity and the most feared complication following PD, the choice of the pancreato-enteric anastomosis technique has been a longstanding subject of debate [5,13]. Recently, a meta-analysis involving 2365 patients who underwent duct-to-mucosa PJ, invagination PJ, or PG found no statistically significant differences among these three techniques in terms of the incidence of CR-POPF, biliary leakage, delayed gastric emptying, in-hospital mortality, internal hemorrhage, or reoperation [13]. In other studies, PJ and PG techniques were similar [14]—or even, in a more recent study, PG was superior compared to PJ concerning CR-POPF rates [15]; however, in both studies PG was associated with a higher rate of postoperative bleeding. Moreover, the multicenter trial “RECOPANC” compared PG and PJ after PD, hypothesizing that PG would result in a lower CR-POPF rate. The results showed no significant differences in major outcomes; however, PG, while less demanding and equally safe, was also associated with a higher rate of postoperative grade A/B hemorrhage [16]. Nonetheless, it is true that no reliable evidence currently favors the use of PJ over PG [8]. All this data reveals that, currently, the evidence supporting whether PJ or PG is still unclear.

It is not only uncertain which type of anastomosis is better, either PJ or PG, but also, within PJ, the anastomotic technique is variable. The Cattell–Warren anastomosis (CWA) is the prototype of duct-to-mucosa anastomosis, which is still practiced in some centers; however, BA is widely used as well [17]. The “PANasta Trial,” a multicenter, double-blind, randomized controlled study, analyzed these two anastomosis techniques and concluded that the BA technique did not reduce complications compared to the CWA [18]. Additionally, a systematic review assessed duct-to-mucosa PJ with other types of PJ techniques (e.g., invagination PJ, binding PJ) in 11 randomized controlled trials. The authors conclude that there is no evidence to support duct-to-mucosa PJ or invagination PJ in terms of the rate of grade B or C POPF, postoperative mortality, rate of surgical reintervention, postoperative bleeding, overall rate of surgical complications, and length of hospital stay. Regarding BA, the evidence is also very uncertain as to whether duct-to-mucosa PJ using the modified Blumgart technique is superior, equivalent, or inferior to duct-to-mucosa PJ using the traditional interrupted technique [19]. On the other hand, a meta-analysis that included twelve studies and 2368 patients suggested that BA may reduce the risk of CR-POPF. As a result, POPF-related complications, including the overall morbidity rate, grade B/C postpancreatectomy hemorrhage, and prolonged postoperative hospital stay, could potentially be decreased by adopting this reconstruction technique [12].

Therefore, most literature indicates that no surgical approach has proven to be superior in completely eliminating the risk of POPF, and its prevention remains a technical challenge for surgeons [18]. Nevertheless, placing an internal or external intrapancreatic trans-anastomotic stent during the surgery in the pancreatic duct is the only factor that has demonstrated to prevent POPF [20,21]. Although these findings exist, the International Study Group of Pancreatic Surgery asserts that there is insufficient high-level evidence to support the use of stents in pancreato-enteric anastomosis to reduce the incidence of POPF [22]. However, some literature suggests that for high-risk patients, particularly those with a soft pancreatic texture and non-dilated pancreatic duct, PJ with an externalized stent may represent the optimal reconstruction technique [23,24,25]. At our center, the presence of these risk factors is not considered a contraindication to performing the BA. In such patients, the placement of an externalized trans-anastomotic stent is routinely employed.

Robotic BA after RPD is considered a safe and simple procedure, offering surgical outcomes that are non-inferior to those of open modified BA [26,27]. When comparing survival outcomes between RPD and OPD, using the same technique for pancreatic stump reconstruction with modified BA, studies have concluded that RPD is associated with less blood loss, reduced delayed gastric emptying, and a higher lymph node yield. Furthermore, the evidence supports that RPD is not inferior to OPD in terms of CR-POPF, surgical risks, and survival outcomes [26].

Ultimately, pancreato-enteric reconstruction using BA after PD is widely accepted in the literature, offering multiple advantages when performed through a robotic approach.

## 5. Conclusions

Robotic Blumgart anastomosis represents a safe and reproducible technique for pancreato-enteric reconstruction after PD. Compared to OPD, RPD provides comparable outcomes, while offering specific advantages such as reduced blood loss and pain due to the minimally invasive approach, as well as lower rates of delayed gastric emptying and a higher lymph node yield, which may be attributed to the enhanced precision and visualization afforded by the robotic platform. Despite the lack of definitive evidence favoring one anastomotic technique over another, the current literature suggests that robotic BA is a safe and effective option, especially when systematically performed and combined with trans-anastomotic pancreatic duct stenting. These findings support the use of the BA and warrant further investigation of this technique in patients undergoing PD.

## Data Availability

The raw data supporting the conclusions of this article will be made available by the authors upon request and can only be shared anonymously.

## Supplementary Materials

The following supporting information can be downloaded at: https://www.mdpi.com/article/10.3390/jcm14134471/s1. Video S1: A step-by-step guide for robotic Blumgart pancreaticojejunostomy.

## Abbreviations

The following abbreviations are used in this manuscript:

PD	Pancreaticoduodenectomy
RPD	Robotic pancreaticoduodenectomy
OPD	Open pancreaticoduodenectomy
PJ	Pancreaticojejunostomy
PG	Pancreaticogastrostomy
POPF	Postoperative pancreatic fistula
CR-POPF	Clinically relevant postoperative pancreatic fistula
BA	Blumgart anastomosis
CWA	Cattell–Warren anastomosis
